# A General Food Chain Model for Bioaccumulation of Ciguatoxin into Herbivorous Fish in the Pacific Ocean Suggests Few *Gambierdiscus* Species Can Produce Poisonous Herbivores, and Even Fewer Can Produce Poisonous Higher Trophic Level Fish

**DOI:** 10.3390/toxins17110526

**Published:** 2025-10-25

**Authors:** Michael J. Holmes, Richard J. Lewis

**Affiliations:** Institute for Molecular Bioscience, The University of Queensland, Brisbane 4072, Australia; r.lewis@uq.edu.au

**Keywords:** ciguatera, ciguatoxin, parrotfish, surgeonfish, depuration, *Gambierdiscus*, *Fukuyoa*, *Scarus*, *Chlorurus*, *Ctenochaetus*, *Naso*, food chain model

## Abstract

We adapt previous conceptual and numerical models of ciguateric food chains for the bioaccumulation of Pacific-ciguatoxin-1 (P-CTX-1) to a general model for bioaccumulation of P-CTX3C by parrotfish (*Scarus frenatus*, *S. niger*, and *S. psittacus*) that feed by scraping turf algae, and surgeonfish (*Naso unicornis*) that mostly feed on macroalgae. We also include the Indian Ocean parrotfish *Chlorurus sordidus* as a model for an excavator feeding parrotfish and include comparisons with the detritivorous surgeonfish *Ctenochaetus striatus* that brush-feeds on turf algae. Our food chain model suggests that, of the *Gambierdiscus* and *Fukuyoa* species so far analysed for ciguatoxin (CTX) production from the Pacific, only *G. polynesiensis* produces sufficient P-CTX3C to consistently produce parrotfish or *N. unicornis* with poisonous flesh. Our model suggests that insufficient CTX would accumulate into the flesh of parrotfish or *N. unicornis* to become poisonous from ingesting benthic dinoflagellates producing ≤0.03 pg P-CTX3C eq./cell, except from extended feeding times on high-density blooms and in the absence of significant depuration of CTX. Apart from *G. polynesiensis*, only *G. belizeanus* and possibly *G. silvae* and *G. australes* are thought to produce >0.03 pg P-CTX3C eq./cell in the Pacific. However, with relatively low maximum concentrations of ≤0.1 pg P-CTX3C eq./cell it is likely that their contribution is minimal. Our model also suggests that the differences between the area of turf algae grazed by parrotfish and similar sized *C. striatus* results in greater accumulation of CTX by this surgeonfish. This makes *C. striatus* a higher ciguatera risk than similar sized parrotfish, either directly for human consumption or as prey for higher trophic level fishes, consistent with poisoning data from Polynesia. It also suggests the possibility that *C. striatus* could bioaccumulate sufficient CTX to become mildly poisonous from feeding on lower toxicity *Gambierdiscus* or *Fukuyoa* species known to produce ≥0.02 P-CTX3C eq./cell. This indicates the potential for at least two food chain pathways to produce ciguateric herbivorous fishes, depending on the CTX concentrations produced by resident *Gambierdiscus* or *Fukuyoa* on a reef and the grazing capacity of herbivorous fish. However, only *G. polynesiensis* appears to produce sufficient P-CTX3C to consistently accumulate in food chains to produce higher trophic level fishes that cause ciguatera in the Pacific. We incorporate CTX depuration into our model to explore scenarios where mildly poisonous parrotfish or *N. unicornis* ingest CTX at a rate that is balanced by depuration to estimate the *Gambierdiscus*/*Fukuyoa* densities and CTX concentrations required for these fish to remain poisonous on a reef.

## 1. Introduction

Ciguatera is a disease caused by eating normally edible tropical and subtropical fishes that have become contaminated through their diet with a class of potent, lipid-soluble toxins called ciguatoxins (CTX) [1]. Ciguatoxins are produced by benthic dinoflagellates that belong to the genera *Gambierdiscus* and *Fukuyoa* [2]. Twenty species of *Gambierdiscus* and four species of *Fukuyoa* have so far been described [2,3,4,5,6]. Benthic dinoflagellates are typically found as epiphytes on turf or macroalgae on coral reefs, although they can also be found on rocky reefs and other substrates [2]. Higher trophic level predatory fish become poisonous from feeding on herbivorous/detritivorous/grazing/scraping/excavating species (hereafter collectively referred to as herbivores) that accumulate CTX by feeding on turf or macroalgae supporting CTX-producing populations of *Gambierdiscus* and/or *Fukuyoa* [7].

The structures of the dominant CTX analogs vary between the major oceans with the Pacific Ocean-CTX (P-CTX) and Caribbean-CTX (C-CTX) being the best characterised [2,8,9,10,11,12]. The structures of the Indian Ocean-CTX (I-CTX) [13,14] have not yet been determined due to yield losses during purification to isolate the toxins(s). Two structural families of CTX dominate in the Pacific, P-CTX-1 (also known as CTX1B) and P-CTX3C (also known as CTX3C) [2]. P-CTX-1 is derived from oxidative biotransformation of P-CTX-4B (CTX4B) and P-CTX-4A (CTX4A, 52-*epi*-CTX4B) produced by some species of *Gambierdiscus* and *Fukuyoa* [2,15,16,17,18]. The biogeographical separation of different CTX analogs produced by various *Gambierdiscus* species is an active area of research with P-CTX3C analogs occurring in ciguateric fishes in the Indian Ocean [19,20] and I-CTX in the Pacific Ocean [21] and more recently the discovery of P-CTX-1, P-CTX-2 (52-*epi*-54-deoxy-P-CTX-1) and P-CTX-3 (54-deoxy-P-CTX-1) in fish from the south western Indian Ocean [20], although the lack of detection of I-CTX from these fish is interesting. Holmes and Lewis [22] have also suggested that, based upon prevailing currents, *Gambierdiscus* producing the P-CTX-1 family of toxins will likely be found in the north-eastern Indian Ocean.

The P-CTX3C family of toxins dominate the CTX profile of cultures of the highest CTX producer known in the Pacific, *G. polynesiensis* [2,23,24,25,26,27,28] and this species is likely the dominant producer of CTX in French Polynesia [24]. It would not be surprising if P-CTX3C-analogs also dominated ciguatera toxin profiles across all of Polynesia, considering that the highest known P-CTX3C concentrations were extracted from *G. polynesiensis* isolated from Rarotonga (Cook Islands, Polynesia) [29,30]. Although CTX concentrations are typically quantified in terms of P-CTX3C equivalents (eq.), the major structural analog found in *G. polynesiensis* is its isomer P-CTX3B (49-*epi*-P-CTX3C) [2,23,24,25,26,27,28], which may be more potent (toxin equivalency factor relative to P-CTX-1 of 0.28 [31]) compared to P-CTX3C (toxin equivalency factor of 0.2 [2]).

Holmes et al. [7] developed conceptual models for the food chain transfer of P-CTX-4 analogs from *Gambierdiscus* into intermediate vectors, and then to the high trophic level fishes that typically cause ciguatera along the east coast of Australia. From these, we derived a numerical model to quantify these transfers in marine food chains into pelagic Spanish mackerel (*Scomberomorus commerson*) in Platypus Bay [32], and benthic grouper (*Plectropomus leopardus*) and parrotfish (*Scarus* spp. and *Chlorurus* spp.) on the Great Barrier Reef [33,34]. These top-down models estimate the population densities of CTX-producing *Gambierdiscus* on turf algae that produce the minimum CTX load to contaminate the flesh of fishes with P-CTX-1 eq. that could cause mild poisoning in humans (0.1 μg P-CTX-1 eq./kg fish) [35]. This CTX concentration is 10-fold more than the precautionary limit of the US FDA [36]. In contrast to our top-down model, Parsons et al. [37] developed a bottom-up model for the Caribbean for *Gambierdiscus* epiphytic on macrophytes and turf algae that incorporated seasonality, variable grazing, and *Gambierdiscus* taxa of varying toxicities. These complementary modelling approaches contextualize the flow of CTX through marine food chains and quantify trophic transfers based upon limited experimental data. Our food chain model is currently based upon linear approximations that are unlikely to be fully representative of nature; however, it allows us to explore hypothetical scenarios and develop testable predictions about the ecology of ciguatera. We do this by using the model to estimate limitations within food chains for the bioaccumulation of CTX to produce ciguateric fishes.

In this paper, we adapt our model for bioaccumulation of P-CTX-1 into food chains on the Great Barrier Reef [32,33,34] to a general model for bioaccumulation of P-CTX3C into parrotfish and the bluespine unicornfish (surgeonfish) *Naso unicornis* that often cause ciguatera across the Pacific Ocean [38,39,40,41,42,43,44]. We suggest that *G. polynesiensis* is the only benthic dinoflagellate species known to produce high enough concentrations of P-CTX3C eq. to consistently produce ciguateric second and higher trophic level fishes in the Pacific. However, the larger area grazed by the lined bristletooth surgeonfish *Ctenochaetus striatus* compared to similar sized parrotfish suggests that this surgeonfish could bioaccumulate sufficient P-CTX3C to become poisonous from ingesting less potent CTX-producing species of dinoflagellate, indicating the potential for at least two food chains to produce ciguateric fishes in the Pacific depending upon the CTX concentrations produced by local *Gambierdiscus* and *Fukuyoa*. We use our model to explore the role of CTX depuration in the development of ciguateric fishes, a factor missing from previous models for ciguateric food chains.

## 2. Results and Discussion

### 2.1. Modelling the Bioaccumulation of P-CTX3C into the Flesh of Parrotfish

**Figure 1:**
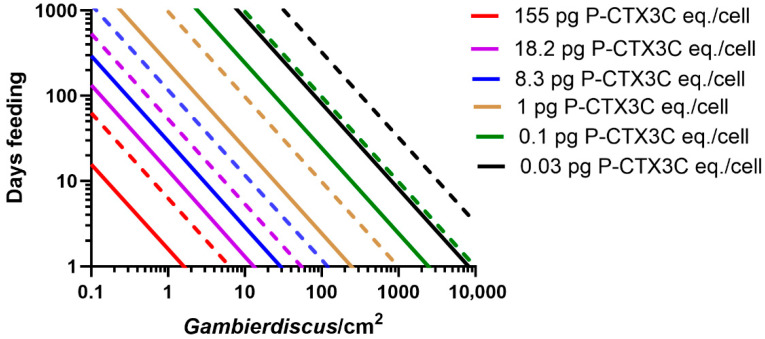
Modelled grazing times (days) for a 25 cm *Scarus frenatus* to accumulate a flesh concentration of 0.5 μg P-CTX3C eq. from feeding on turf algae supporting different densities of *Gambierdiscus* producing 0.03–155 pg P-CTX3C eq./cell. The model produces two outcomes for each cell concentration depending upon whether 10% or 40% of the toxin-load ingested accumulates into flesh: solid line = 40%, dashed line = 10% for each P-CTX3C concentration. Scenarios are based on bioaccumulation of CTX without depuration and ignore the ‘CTX dilution’ effect of fish growth over longer feeding times [7].

**Figure 2:**
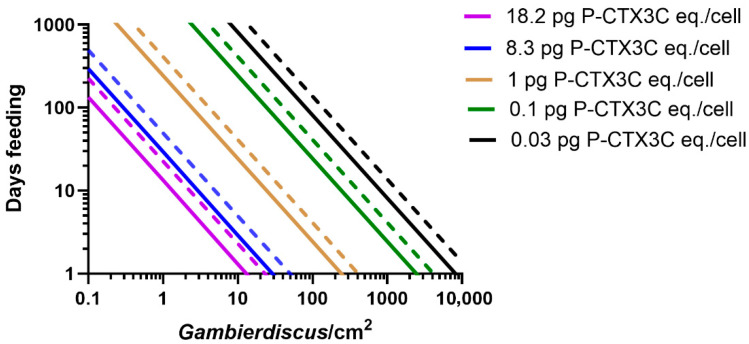
Comparison of modelled grazing times (days) for a 25 cm *Scarus frenatus* (solid line) and *S. niger* (dashed line) to accumulate a flesh concentration of 0.5 μg P-CTX3C eq. from feeding on turf algae supporting different densities of *Gambierdiscus* producing 0.03–18.2 pg P-CTX3C eq./cell. The model produces two outcomes for each cell concentration depending upon whether 10% or 40% of the toxin-load ingested accumulates into flesh: only 40% (maximum potential toxicity) plotted for each P-CTX3C concentration. Scenarios are based on bioaccumulation of CTX without depuration and ignore the ‘CTX dilution’ effect of fish growth over longer feeding times [7].

**Figure 3:**
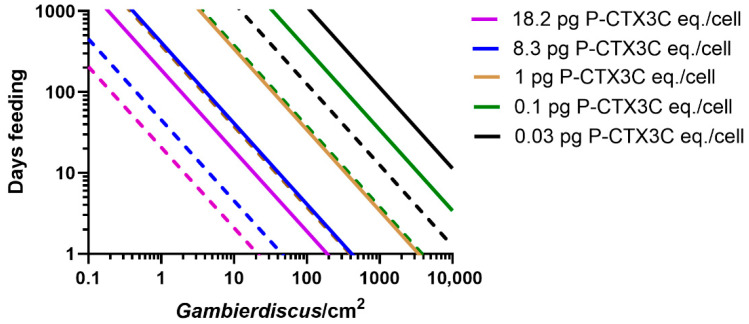
Comparison of modelled grazing times (days) for a 25 cm *Scarus psittacus* (solid line) and *Chlorurus sordidus* (dashed line) to accumulate a flesh concentration of 0.5 μg P-CTX3C eq. from feeding on turf algae supporting different densities of *Gambierdiscus* producing 0.03–18.2 pg P-CTX3C eq./cell. The model produces two outcomes for each cell concentration depending upon whether 10% or 40% of the toxin-load ingested accumulates into flesh: only 40% (maximum potential toxicity) plotted for each P-CTX3C concentration. Scenarios are based on bioaccumulation of CTX without depuration and ignore the ‘CTX dilution’ effect of fish growth over longer feeding times [7].

We model the minimum number of days that 25 cm *Scarus frenatus*, *S. niger*, *S. psittacus*, and *Chlorurus sordidus* would need to feed on turf algae supporting *Gambierdiscus* producing known concentrations of P-CTX3C eq./cell (Figure 1, Figure 2 and Figure 3). The model suggests that parrotfish of this size would have to feed continuously for >8 months on turf algae supporting 10 *Gambierdiscus*/cm^2^ producing 0.1 pg P-CTX3C eq./cell to accumulate sufficient CTX to become mildly poisonous (Figure 1, Figure 2 and Figure 3). In nature, the density of epiphytic dinoflagellates on turf algae will vary in both space and time and often be composed of a mix of species [24,45,46] with densities on mesh screen assays mostly <10 cells/cm^2^, and a global median density of ~1 cell/cm^2^ [47]. A scenario where fish feed for >8 months on 10 *Gambierdiscus*/cm^2^ producing 0.1 pg P-CTX3C eq./cell that results in a poisonous fish (Figure 1, Figure 2 and Figure 3) is unlikely, since our model is based upon continuous bioaccumulation of CTX in the absence of depuration. Any amount of depuration would require greater CTX loads to be ingested to produce a poisonous parrotfish. Our model uses a 25 cm total length fish, which is relatively small for these parrotfish species, which can grow to larger sizes; however, small sized fish are often eaten throughout the Pacific, with 23 and 24 cm, respectively, being the average size of *Scarus* and *Chlorurus* spp. eaten on Moorea in French Polynesia [44]. Larger sized fish would have to accumulate greater CTX loads to produce poisonous flesh [34].

A cell concentration of 0.1 pg P-CTX3C eq./*Gambierdiscus* is equal or greater than the maximum CTX concentration so far reported for most of the *Gambierdiscus* species that occur in the Pacific Ocean (Table 1). The presence of these low CTX-producing species on reefs would contribute small CTX loads to parrotfish food chains but, in the absence of major blooms, their overall contribution to the risk of producing ciguateric fishes is likely minimal, unless unknown environmental conditions can greatly stimulate their production of CTX. Based on our model (Figure 1, Figure 2 and Figure 3), it is likely that only *Gambierdiscus* species that produce >0.1 pg P-CTX3C eq./cell are major contributors to the production of ciguateric parrotfishes.

*Gambierdiscus polynesiensis* is the only species from the Pacific Ocean that so far appears capable of producing sufficient P-CTX3C to consistently produce a poisonous 25 cm parrotfish (Figure 1, Figure 2 and Figure 3, Table 1), although several *Gambierdiscus* and *Fukuyoa* species are yet to be analysed for CTX concentrations (Table 1), and some species should be reanalysed using updated methods for toxin detection and quantification. *Gambierdiscus belizeanus* and possibly *G. silvae* and *G. australes* may sometimes also produce sufficient CTX (Table 1) to produce poisonous parrotfish in the Pacific but only if the parrotfish graze on combinations of high-density blooms with long feeding times (Figure 1, Figure 2 and Figure 3), with even greater combinations of CTX concentrations and/or grazing times necessary if fish simultaneously depurate CTX. For example, a 25 cm *S. frenatus* would have to graze on 100 *G. belizeanus*/cm^2^ producing its maximum known CTX concentration of 0.1 pg P-CTX3C eq./cell (Table 1) for 24–97 days to become mildly poisonous in the absence of any significant simultaneous depuration (Figure 1). It is likely that only a small number of *Gambierdiscus* and *Fukuyoa* species are responsible for producing ciguateric parrotfish across the Pacific with *G. polynesiensis* a principal cause. Chinain et al. [24] previously suggested that *G. polynesiensis* was likely the dominant producer of CTX in French Polynesia and Murray et al. [6] recently suggested that *G. polynesiensis* might be responsible for most of the ciguatera throughout the South Pacific. The relatively high toxicity of *G. excentricus* (maximum ~0.5–2.6 pg P-CTX3C or P-CTX-1 eq./cell) from the Atlantic [48,55,56] suggests that this species and *G. polynesiensis* are the two species thus far known capable of producing sufficient CTX to cause much of the ciguatera around the globe, although the origin of any CTX-contaminating ciguateric fish across the eastern and western Atlantic remains to be proven.

*Scarus frenatus*, *S. niger*, and *Ch. sordidus* could become poisonous (in the absence of significant depuration) from feeding for <20 days (Figure 1, Figure 2 and Figure 3) on turf algae supporting 10 *G. polynesiensis*/cm^2^ producing the maximum known CTX concentration from French Polynesian isolates (8.3 pg P-CTX3C eq./cell) [27]. However, *S. Psittacus* would require longer feeding times to become poisonous (Figure 3), because of the apparently much smaller area grazed relative to other parrotfish species [61]. The highest CTX concentration produced by *Gambierdiscus* has generally been thought to be 18.2 pg P-CTX3C eq./cell [2] from a *G. polynesiensis* isolate from Rarotonga in the Cook Islands, Polynesia [29]. However, this isolate (CAWD212) reportedly produced concentrations as high as 155 pg CTX/cell [30]. If *G. polynesiensis* can produce such high CTX concentrations in nature, then a 25 cm *S. frenatus* could become poisonous by feeding for <1 day on 10 cells/cm^2^ (Figure 1).

*Gambierdiscus polynesiensis* appears to have a broad geographical distribution, being found in more regions as more studies are conducted, including recently from both the eastern and western margins of the Coral Sea [6,62,63]. The first detection of *G. polynesiensis* from Australia (western Coral Sea) was from a DNA-metabarcoding study of the contents of the pharyngeal mill of the surf parrotfish, *S. rivulatus*, from the northern Great Barrier Reef [62] with a broad north–south distribution along the Great Barrier Reef recently confirmed by Murray et al. [6]. Detection of *G. polynesiensis* from the gut contents of two *S. rivulatus* [62] is the first confirmation that parrotfish ingest *Gambierdiscus* and only the third confirmation of herbivorous fish feeding on *Gambierdiscus* in the wild, the first two being cells found in the gut contents of surgeonfish from French Polynesia [64] and Hawaii [65]. Future DNA-metabarcoding studies on the gut contents of herbivorous coral reef fishes could help confirm the distribution of benthic dinoflagellate species consumed by herbivores in global marine food chains and provide insights into the workings of regional food chains.

*Gambierdiscus polynesiensis* is the only species confirmed to produce the precursors of P-CTX-1 (P-CTX-4A and -4B) in the Pacific [2,16,18,25,27,66,67]. Although P-CTX-1 analogs have also been suggested to be produced by an isolate of *F. paulensis* from the Mediterranean Sea [58], no ciguateric fishes have been confirmed from this area. In some regions of the Pacific, P-CTX-1 analogs are the dominant CTX in ciguateric fishes, such as the east coast of Australia [68,69,70] and Kiribati [9,71,72,73]. However, combinations of the P-CTX-1 and P-CTX3C families of toxins can cooccur in ciguateric fishes from French Polynesia [24], Japan [67] and Fiji [74], with analogs of both families of toxins also found separately from ciguateric fishes in Japan [75,76,77]. As yet, there are no toxicity studies of *G. polynesiensis* from the east coast of Australia, but it is likely that such studies will find P-CTX-1-analogs as the dominant toxins, with some isolates possibly producing up to 1.6 pg P-CTX-1 eq./cell [33]. The recent discovery of *G. polynesiensis* from the west coast of New Caledonia (eastern Coral Sea) supports the dominance hypothesis of the P-CTX-1 family of toxins in this region, as only P-CTX-4 analogs could be detected from this isolate, although the concentrations were low (~0.01 and 0.02 pg P-CTX3C eq./cell for P-CTX-A and -B, respectively) [63].

Our previous models for fishes on the Great Barrier Reef generally limited interpretation of the bioaccumulation of CTX into herbivores over ≤1 month because high grazing rates across the Reef, combined with slow growth rates for *Gambierdiscus* [33,34], likely reduce the opportunities for *Gambierdiscus* blooms to develop. This may not be the case where herbivorous fishes are heavily targeted for food and where fishing pressure may be high enough to reduce grazing pressure, allowing for larger *Gambierdiscus* populations to grow. Although biomass is an imperfect predictor of ecological processes, Pacific coral reef islands with the lowest herbivore biomass typically also have the lowest rates of herbivory [78]. It is interesting that only low cell densities have so far been found from the Great Barrier Reef, with the only blooms so far found along the east coast of Australia occurring in temperate waters well south of the Great Barrier Reef [79,80]. In such circumstances, it may be only the higher CTX-producing species such as *G. polynesiensis* responsible for producing sufficient CTX load to occasionally cause ciguatera on the Great Barrier Reef.

### 2.2. Modelling the Bioaccumulation of P-CTX3C into the Flesh of Naso Unicornis

Unicornfish (*Naso* spp.) are surgeonfish that mostly feed on macroalgae, with *N. unicornis* mostly feeding on phaeophytes such as *Sargassum* spp. (Ochrophyta) [81,82,83,84], although some *Naso* also have been observed to feed on turf algae [85]. Despite its potential to cause ciguatera [40,41,44], *N. unicornis* is a highly desired food fish across Polynesia where it is often targeted by spearfishing at night [44,85,86,87,88] with 28 cm being the average size unicornfish eaten in Moorea, French Polynesia [44]. The difficulty in developing food chain models for the bioaccumulation of CTX into fishes that graze macroalgae has been the paucity of data on the weight of algae consumed by herbivores per unit of time (which likely varies seasonally [89]). If such data were available, it would be easy to couple them with the most common (simplest) method for estimating *Gambierdiscus*/*Fukuyoa* populations in the wild, i.e., cells/g wet weight macroalgae [2,7], at least for those herbivores that graze on macroalgae. However, the diverse and complex morphologies of macroalgae (filamentous and thallus) mean that the surface area supporting epiphytic benthic dinoflagellates relative to the weight of algae are not comparable between species, which was a major reason for the development of artificial screen assays by Tester et al. [47,90]. Parsons et al. [37] were able to simulate seasonal grazing rates/m^2^ for sites in the Caribbean to produce broad-scale estimates of CTX loads being ingested by the community of second trophic level organisms. However, we have chosen to model the daily consumption of macroalgae by herbivorous fish as a percentage of the fish’s bodyweight (10–30%) as this sets conservative limits for the maximum number of *Gambierdiscus* consumed/day and hence the potential CTX load accumulated by these herbivores grazing algae with different densities of *Gambierdiscus*/*Fukuyoa* producing different concentrations of CTX.

**Figure 4:**
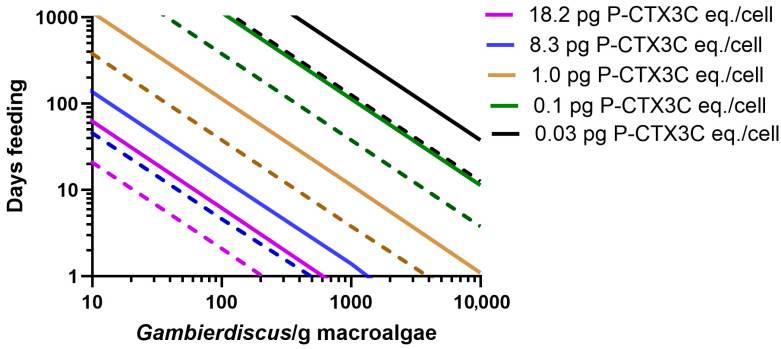
Modelled grazing times (days) for a 28 cm *Naso unicornis* to accumulate a flesh concentration of 0.5 μg P-CTX3C eq. from feeding on macroalgae supporting various densities of *Gambierdiscus* producing 0.03–18.2 pg P-CTX3C eq./cell. The model is based on the weight (g) of macroalgae eaten/day as a percentage of the fish bodyweight with 44% of ingested CTX partitioning into flesh [92]. Solid line = 10% bodyweight (28 g) of macroalgae eaten/day. Dashed line = 30% bodyweight (85 g) of macroalgae eaten/day. Scenarios are based on bioaccumulation of CTX without depuration and ignore the ‘CTX dilution’ effect of fish growth over longer feeding times [7].

Our model suggests that it would take between >1–3 y of continuous feeding on 100 *Gambierdiscus*/g macroalgae producing 0.1 pg P-CTX3C eq./*Gambierdiscus* for the flesh of a 28 cm *N. unicornis* to become mildly poisonous (in the absence of significant depuration of CTX) (Figure 4). This seems unlikely and is consistent with our suggestion that other herbivores (parrotfish) would also be unlikely to develop poisonous flesh from grazing on turf algae hosting *Gambierdiscus* that produce ≤0.1 pg P-CTX3C eq./cell, with the two types of models (fish feeding on turf algae or macroalgae) based upon calculations for food chains where the flow of CTX occurs by different vectors. In contrast, a 28 cm *N. unicornis* could develop mildly poisonous flesh from feeding for only 5–14 days on 100 *Gambierdiscus*/g macroalgae producing the maximum known concentration of 8.3 pg P-CTX3C eq./cell from *G. polynesiensis* from French Polynesia [27] (in the absence of significant CTX depuration) (Figure 4). In total, 85% of the average global density estimates for *Gambierdiscus* are <1000 cells/g macroalgae, although this is an underestimate as the literature is biased towards the reporting of high cell densities [91]. Our model suggests that unicornfish could become mildly poisonous from feeding on a bloom of 1000 *Gambierdiscus*/g macroalgae producing 1.0 pg P-CTX3C eq./cell in as little as 4–12 days (Figure 4).

Although based upon different food chains, the models for bioaccumulation of CTX into parrotfish and unicornfish suggest that neither group of fishes is likely to become poisonous from feeding on algal hosts, supporting *Gambierdiscus* that produce ≤0.1 pg P-CTX3C eq./cell (Figure 1, Figure 2, Figure 3 and Figure 4), unless they feed continuously for extended periods of time on high-density blooms and in the absence of any significant depuration of CTX. An advantage of estimating ingested CTX loads based upon percentages of bodyweight/day is that the model results are no longer dependent on the inherent uncertainties estimating the hours that fish spend feeding/day, or the area of turf algae grazed by parrotfish/day.

### 2.3. The Species of Herbivore Feeding on Gambierdiscus Affects the Risk of Ciguateric Fishes Being Produced in Food Chains

**Figure 5:**
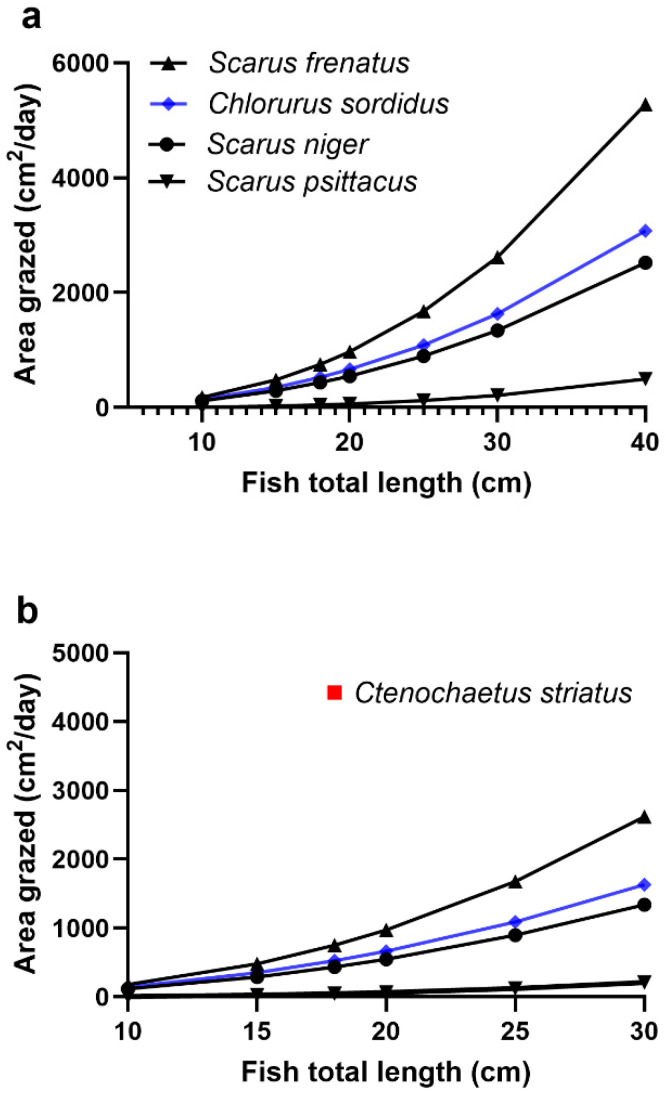
Area of turf algae grazed per day by parrotfish and the lined surgeonfish *C. striatus*. (**a**) Area grazed by 4 species of parrotfish, 3 scraper (black lines) and 1 excavator (blue line) species. (**b**) Comparison with 18 cm *C. striatus* (red square).

The area grazed by feeding parrotfish is a function of their size [34]. We estimated the area grazed by four parrotfish species, three scraper and one excavator species with *S. frenatus* grazing the largest area and *S. psittacus* the least (Figure 5a). Our previous parrotfish model for ciguateric food chains on the Great Barrier Reef was based on *S. niger* [34], which grazes an intermediate area between these two other scraping species (Figure 5a). The larger the area grazed, the more *Gambierdiscus* could potentially be ingested, accumulating a larger CTX load. However, the potentially greater CTX load incorporated into the proportionately greater body mass of larger fish can lead to a lower CTX concentration in their flesh ([34] and references therein). Herbivore species graze different habitats across reefs [93,94], with these differences sometimes pronounced [95,96]. Indeed, recent research suggests that some parrotfishes prefer to feed on turf algae hosting dinoflagellates and other protein-rich photoautotrophic organisms [96]. During the day, *S. psittacus* and *S. niger* are believed to predominantly feed over the deeper slope regions of reefs compared to *S. frenatus* and the surgeonfish *C. striatus*, that are often abundant near the reef crest [93,97,98]. Benthic dinoflagellate populations can show considerable spatial and temporal variation [24,45,79,99,100,101,102,103], but most available data do not discriminate between species, with no data where populations of *G. polynesiensis* predominate across reef profiles/microhabitats and if specific combinations of habitat and conditions favour growth. However, given how frequently *C. striatus* is associated with ciguatera across the South Pacific [40,43,99,104], it is likely that its feeding area often overlaps with the distribution of ciguatera-producing dinoflagellates. Future research should consider the distribution and population dynamics of high-risk *Gambierdiscus* species across reefs, especially in relation to the feeding ranges of herbivorous fishes.

The area of turf algae grazed daily by parrotfish appears to be much smaller than that of an equivalent sized *C. striatus*, with the surgeonfish potentially grazing ~4–5-fold more area of turf algae compared to *S. niger*, *S. frenatus*, and *Ch. sordidus* (Figure 5b). These estimated differences are likely conservative given the assumptions used by our model for grazing efficiency (as no data is available). Our model uses a 90% grazing efficiency for parrotfish ingesting *Gambierdiscus* but only a 50% efficiency for *C. striatus* ([33,34], this paper). This is because we consider the scraping action of parrotfish likely more effective at removing epiphytes [34] than the brushing mode of feeding by *C. striatus* [33]; however, this requires further research to determine the actual feeding efficiencies. In addition, an equivalent length (18 cm) *S. niger* (~116 g) or *S. frenatus* (~126 g) [105] is likely slightly heavier than *C. striatus* (~101 g) [106,107], suggesting the toxin concentration per g could be higher in the surgeonfish. The much smaller area grazed by parrotfish relative to *C. striatus* is likely because parrotfish ingest much larger amounts of material with each bite than *C. striatus* and take much fewer bites to fill their stomach [108]. In addition, the jaw gape of *C. striatus* can open to nearly 180^o^, biting a large area relative to their size [97,109]. The long retention time for food material in the gut of *C. striatus*, twice that of some parrotfish [108], may also be a factor in determining the amount of CTX absorbed by *C. striatus* relative to parrotfish.

The larger area grazed by *C. striatus* relative to parrotfishes (Figure 5) may be why this surgeonfish is one of the first herbivores to become poisonous [40] and one of the most frequently poisonous fish found on South and Central Pacific reefs [40,43,99,104]. Ciguatera became a major problem in Rarotonga in the 1980s [40] and is coincidentally where the most potent known strain of *G. polynesiensis* was isolated [29,30]. Rongo and van Woesek [40] described the development of ciguatera in Rarotonga as occurring in three phases, with ciguateric fish in the first phase (1989–2000), primarily *C. striatus* and to a lesser extent *N. unicornis*, indicating the likely presence of CTX-producing *Gambierdiscus* on both turf algae and macroalgae. *Ctenochaetus striatus* have broad distributions across reefs [110] but are often dominant in shallower areas of upper reef slopes and crests [97,111,112]. If the suggestion that *G. polynesiensis* is the principal cause of ciguatera in the Pacific is correct, then during this first phase in Rarotonga, blooms of *G. polynesiensis* may have occurred in the shallow reef areas that overlap the grazing areas for *C. striatus*. Bagnis et al. [99] earlier demonstrated that the flesh toxicity of *C. striatus* often paralleled changes (both increases and decreases) in *Gambierdiscus* populations at three sites in French Polynesia. Although not recognized at the time, the reductions in toxicity of *C. striatus* that occurred as *Gambierdiscus* populations decreased [99] may be the first indirect evidence for depuration of CTX by *C. striatus*.

During the second phase of ciguatera development (2001–2005), many atypical species caused ciguatera including giant clams (*Tridacna maxima*), goatfish (*Mulloidichthys flavolineatus*), and mullets, especially *Crenimugil crenilabis* (possibly now *Moolgarda* cf. *crenilabis* [113]) [40]. Poisonous giant clams also occur in French Polynesia [24] and suggest that considerable numbers of *Gambierdiscus* must sometimes occur in the water column to be ingested by these filter feeders. In addition, considerable numbers of *Gambierdiscus* must sometimes occur near or within the soft/sandy sediments that feeding mullet and goatfish sift through for food [114]. Mullet (*Mugil cephalus*) have been suggested to depurate CTX with a half-life of ~4 h [115]. If rapid depuration also occurs in the two mullet species causing ciguatera in Rarotonga [40], then this suggests that people were poisoned from eating mullet that had only recently accumulated CTX in the preceding days. It also suggests that for a fast-depurating species to accumulate sufficient CTX to become poisonous, the toxin must have originated from a high CTX producer, such as *G. polynesiensis*. Some of the highest fish CTX concentrations found from ciguatera surveys in French Polynesia were also attributed to mullet (*Crenimugil crenilabis*) [24]. Cooper [116] previously reported that mullets and goatfishes were some of the first fishes to become safe to eat as toxicity declined on reefs in the Gilbert Islands (Kiribati), consistent with faster rates of CTX depuration than many of the carnivorous fish species eaten.

During the third phase (2006–2009), more carnivorous fishes caused poisonings but also at least two parrotfish species, including *S. psittacus*, which, based upon a survey of Rarotonga residents, may have caused more cases of ciguatera than any other fish species [40]. This is surprising given the apparently small area grazed by this species relative to other parrotfish (Figure 5), suggesting it should take considerably longer for *S. psittacus* to accumulate CTX, although the relationship between fish length and area grazed was not significant for this species [61]. This suggests high-density blooms of *G. polynesiensis* with high CTX concentrations (Figure 3) must have been occurring where *S. psittacus* was feeding during this third phase. In Hawaii, *S. psittacus* is mostly found >6 m depth [98], possibly suggesting highly toxic blooms of putative *G. polynesiensis* were occurring in deeper water during this third phase. *Scarus psittacus* has also been reported to accumulate CTX to poisonous levels in Raivavae lagoon, French Polynesia (39).

### 2.4. More than One Food Chain Can Produce Poisonous Herbivores but Only High CTX-Producing Benthic Dinoflagellates Likely Cause Poisonous Carnivores

The 4–5-fold larger area of turf algae grazed by *C. striatus* relative to parrotfish suggests that this surgeonfish could ingest >4-fold more CTX load if they grazed on similar densities of *Gambierdiscus* producing the same cell CTX concentrations. This could effectively reduce the cellular CTX concentration on turf algae required to produce mildly poisonous surgeonfish relative to the same sized parrotfish. If only CTX concentrations of >0.1 P-CTX3C eq./cell likely produce poisonous parrotfish (Figure 1, Figure 2 and Figure 3), it is possible that 4–5-fold lower concentrations (≥0.02 pg P-CTX3C eq./cell) could be sufficient to produce mildly poisonous *C. striatus*, although this needs to be tested. This suggests that 8–9 *Gambierdiscus* and 1 *Fukuyoa* species (Table 1) could potentially be capable of causing mildly poisonous *C. striatus* in the Pacific, although the actual outcome will also depend on the rates of CTX depuration by different surgeonfish species. This has relevance for much of the Pacific where herbivorous reef fish are often a staple of peoples’ diet [43,44,85,86,87,88,117,118,119]. However, it suggests the potential for at least two food chain pathways to produce poisonous herbivorous fish in the Pacific depending on the CTX concentration produced by benthic dinoflagellates, with parrotfish having to ingest higher CTX loads than *C. striatus* to become poisonous.

Although our analysis suggests that less toxic species of *Gambierdiscus* and *Fukuyoa* could contribute to production of poisonous herbivorous fishes across the Pacific, the toxin losses/dilution that occur through trophic transfers and in the partitioning between fish tissues described by our model ([32,33,34], this paper) suggests that they do not produce sufficient toxin for carnivorous fishes to accumulate enough CTX in their flesh to cause human poisoning (Table S1). Our model incorporates three processes that effectively dilute the CTX load transferred and bioaccumulated into third trophic level predators: (1) losses in the transfer between trophic levels (losses during capture, eating, and digestion of prey fish), (2) dilution of CTX concentration in the larger weight of the predator, and (3) portioning the ingested toxin load into the flesh of the predator (Table S1). A scenario calculating how many mildly poisonous, optimal-sized prey fish a 2 kg grouper (e.g., *Plectropomus leopardus*) would have to feed on to produce flesh that is also mildly poisonous [33] suggests consumption of between 60–238 prey (Table S1). Assuming grouper feed three times/week on optimal-sized prey (5% bodyweight [120] = average of 2.1% bodyweight/day), it would take between 140–560 days for the grouper to accumulate sufficient CTX to develop mildly poisonous flesh, assuming that the grouper fed exclusively on mildly poisonous prey and in the absence of any concurrent CTX depuration (Table S1), which seems unlikely. Groupers are opportunistic predators that have been found to feed on fish from up to 15 different families, as well as crustaceans and molluscs [121,122], with any feeding on less-toxic prey lengthening the time for the grouper to become poisonous. It is likely only food chains that include high CTX-producing benthic dinoflagellates produce poisonous carnivores (≥3rd trophic level fishes). Only *G. polynesiensis* in the Pacific (and possibly *G. excentricus* in the Atlantic) are currently known to be able to produce the high CTX concentrations that likely lead to the poisonous higher trophic level fishes that cause ciguatera. However, in subsistence societies and/or those with strong cultural attachment to food from the sea, the risk of ciguatera poisoning may be enhanced by also sourcing herbivorous reef fish for food that may become mildly poisonous from feeding on lower-toxicity *Gambierdiscus* or *Fukuyoa*. It would be an interesting experiment to repeatedly feed predatory fish exclusively on a diet of 0.5 μg P-CTX3C eq./kg food to determine if they can accumulate sufficient CTX to develop flesh contaminated with ≥0.5 μg P-CTX3C eq. within time frames likely to occur in nature. To date, there is no experimental evidence for the transfer of ≥50% of CTX load between trophic levels or for the portioning of ≥50% of ingested CTX load into fish filets.

It appears that the only way the 8–9 lowly ciguatoxic species of *Gambierdiscus* and 1 *Fukuyoa* species (Table 1) can contribute significantly to production of higher trophic level fishes is if they develop very large blooms or if unknown environmental conditions can stimulate these species to produce 10–30-fold higher P-CTX3C eq. concentrations. The maximum CTX concentration so far reported is 155 pg CTX/cell from a *G. polynesiensis* isolate (CAWD212) from the Cook Islands in Polynesia, with 65% of the CTX being P-CTX3B (attributed to unpublished data of the early toxicity of the cultures, Rhodes et al. [30]). The same isolate was later found to produce 18.2 pg P-CTX3C eq./cell [29] and subsequently 0.4 pg total P-CTX eq./cell with 65% still composed of P-CTX3B, but with P-CTX-4A and -4B also detected [52]. This reduction in toxicity that occurs over time in dinoflagellate cultures is not unusual. We do not know if there are environmental stimuli that can produce the opposite effect and increase the toxicity of cells to match the higher CTX concentrations quantified from cultures recently isolated from the wild. The maximum increase in CTX concentration so far found through experimental manipulations of cultured strains is only ~2–3-fold [23,27,123,124].

### 2.5. Depuration: The Missing Link of Food Chain Models

**Figure 6:**
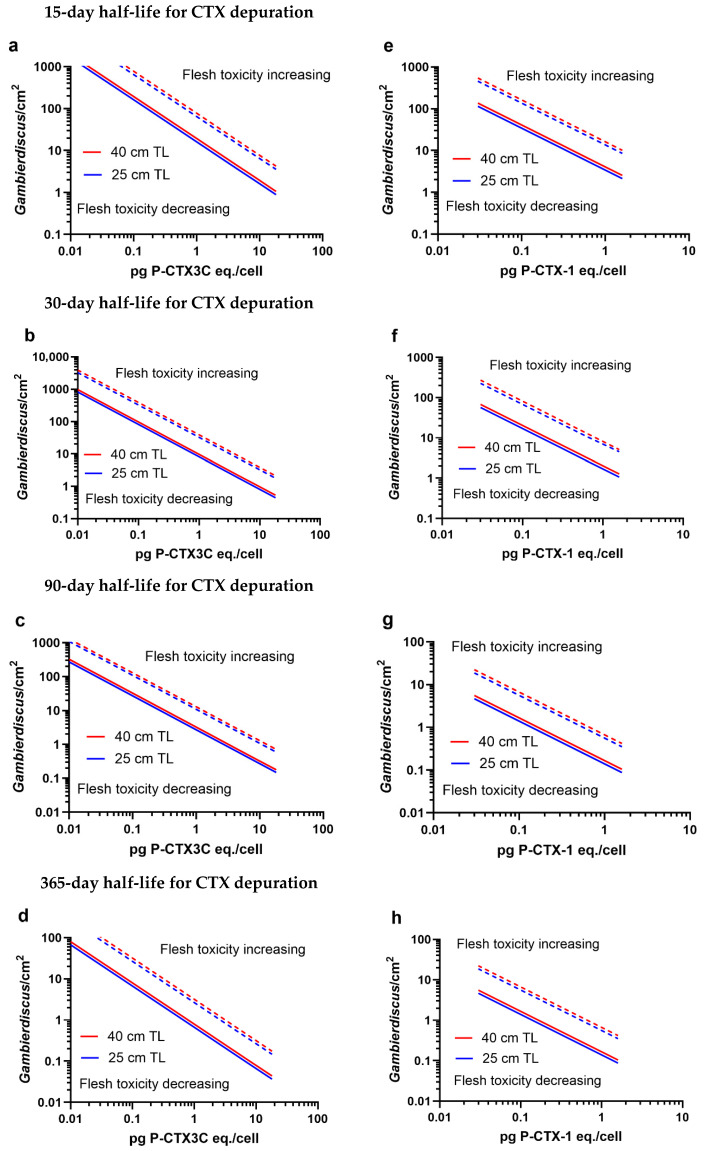
Modelled *Gambierdiscus* densities and P-CTX concentrations that parrotfish (*S. frenatus*) need to continuously feed on to maintain mildly poisonous flesh in 25 and 40 cm fish against CTX loss from hypothetical depuration half-lives of 15, 30, 90, or 365 days. The model produces two outcomes for each cell concentration depending upon whether 10% (dashed line) or 40% (solid line) of the toxin-load ingested accumulates into flesh. (**a**–**d**) *Gambierdiscus* producing 0.01–18.2 pg P-CTX3C eq./cell. (**e**–**h**) *Gambierdiscus* producing 0.03–1.6 pg P-CTX-1 eq./cell.

Depuration of CTX is likely a major factor determining the development and maintenance of ciguateric fishes in food chains [7,15,125,126]. We consider depuration in its broadest sense, encompassing both toxin excretion as well as metabolism to less toxic forms. Herbivorous fish become poisonous when they accumulate sufficient CTX load from ingesting many possible combinations of *Gambierdiscus* densities and CTX concentrations (Figure 1, Figure 2, Figure 3 and Figure 4). A fish may accumulate sufficient CTX to become poisonous, but it must be captured and eaten to cause human poisoning. If the fish depurates CTX before being captured, it will lose toxicity over time and eventually become non-poisonous unless it continues to ingest a CTX load equal to or greater than the losses from depuration. A larger sized fish (40 cm) must ingest a greater CTX load to remain poisonous compared to a smaller fish (25 cm) of the same species, although the difference appears relatively small across a range of CTX concentrations and depuration rates (Figure 6).

**Figure 7:**
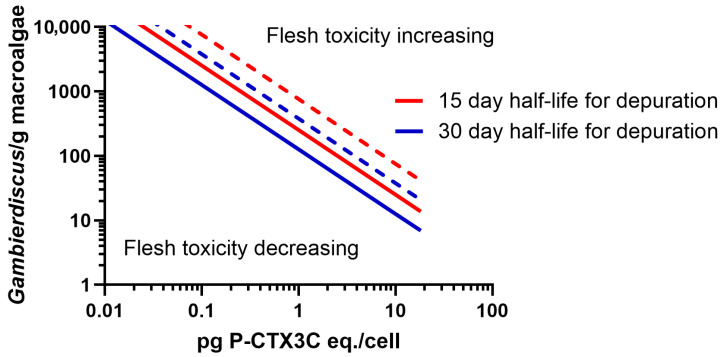
Modelled *Gambierdiscus* densities and P-CTX concentrations (0.01–18.2 pg P-CTX3C eq./cell) that a 28 cm *N. unicornis* needs to continuously feed on to maintain mildly poisonous flesh against CTX loss from hypothetical depuration half-life’s of 15 or 30 days. Weight of macroalgae eaten per day estimated as a percentage of fish bodyweight: 10% = dashed line, 30% = solid line.

As expected, faster rates of CTX depuration require fish to consume increasing CTX loads to remain poisonous (Figure 6 and Figure 7). Our model suggests that a hypothetical CTX depuration half-life of ≤30 days for a 25 cm *S. frenatus* ingesting a *Gambierdiscus* density of ≤1 cell/cm^2^ producing ≤4.5 pg P-CTX3C eq./cell (or ≤1.6 pg P-CTX-1 eq./cell) would result in insufficient CTX load being accumulated for the fish to remain poisonous (Figure 6b,f). In such a scenario, the fish would eventually become non-poisonous, given enough time for depuration to reduce the CTX flesh concentration below that of 0.5 μg P-CTX3C eq./kg or 0.1 μg P-CTX-1 eq./kg. A cell density of ~1 *Gambierdiscus*/cm^2^ is the median density for *Gambierdiscus* from screen assays globally [47] and 4.5 pg P-CTX3C eq. the average CTX concentration of French Polynesian *G. polynesiensis* used by Clausing et al. [92,127] to examine the accumulation of CTX in herbivorous fish. As *S. frenatus* grazes a larger area than the three other parrotfish species modelled (Figure 5), this suggests that these other species would need to ingest even greater CTX loads to remain poisonous. However, if *Gambierdiscus* can produce 155 pg CTX/cell [30], even by a minority of cells within a community of epiphytic benthic dinoflagellate species [24,46], it would likely lead to numerous poisonous fish across several trophic levels, with sufficient toxin load being produced to maintain toxicity against rapid rates of CTX depuration. Conceptually, this could be a reason for the development and maintenance of some ciguatera ‘hot spots’, where fish are frequently poisonous over prolonged periods of time. However, in nature, *Gambierdiscus* populations fluctuate through time [46,99,100,101]. For this reason, ciguatera outbreaks have frequently been attributed to *Gambierdiscus* blooms, although often without considering the toxin load these cells must produce to cause a poisonous fish. Once the bloom is over, the toxicity of the herbivorous fish feeding over the reef will likely reduce depending upon residual CTX loads, with the rate of CTX depuration being a major factor determining how long fish remain poisonous.

As yet, we have no experimental evidence for the depuration half-life of CTX in parrotfish or surgeonfish. However, depuration rates may not be constant, with some evidence for them being concentration-dependent [92]. We need more estimates for the rates of CTX assimilation and depuration in different fish species to build better food chain models, with estimates for *C. striatus* likely being especially useful for the Pacific. While carnivorous fishes were mostly suggested to retain toxicity for many years as the overall toxicity declined on reefs in the Gilbert Islands, herbivorous *Ctenochaetus* spp. (and *Acanthurus xanthopterus*) were also reported to be among those fishes that retained toxicity [116], possibly indicating slow rates of CTX depuration for these surgeonfishes. The current estimates for CTX depuration are based on indirect evidence in carnivorous moray eels and Spanish mackerel, <1 y [7,126], experimental evidence for carnivorous pinfish, 3–5 months [128], carnivorous grouper, ≤1 month for P-CTX-1, -2 and -3 in muscle [129], ~1 month for P-CTX-1 in goldfish muscle (based on Figure 2 in [130]), and hours for detritivorous mullet [115]. Carnivorous lionfish also depurate CTX relatively quickly [131]; however, we could not estimate a half-life for the rate of depuration from the available data. Clausing et al. [92] reported that only 26% of CTX ingested by the unicornfish *Naso brevirostris* assimilated into fish tissues after 120 days of continuous weight-adjusted feeding of *G. polynesiensis*. This is much less than the 43% our model would have estimated in the absence of any depuration (the model incorporates a 43% transfer efficiency across trophic levels (this paper, [128])). We do not know the mechanism that determines the amount of toxin retained by any fish species over time; however, Clausing et al. [92] suggest that the quantity of CTX retained by a fish is proportional to that consumed. The 26% of toxin retained by *N. brevirostris* over 120 days of weight-adjusted feeding of *G. polynesiensis* [92] is consistent with a half-life for toxin depuration of <15 days (Table S2, Figure S1).

A hypothetical rapid depuration half-life of 15 days would result in a mildly poisonous *S. frenatus* (0.5 μg P-CTX3C eq./kg flesh) having to continuously feed on >15 *Gambierdiscus*/cm^2^ producing ≥1 pg P-CTX3C eq./cell to remain poisonous (Figure 6a). *Gambierdiscus polynesiensis* is the only species thus far known capable of producing ≥1 pg P-CTX3C eq./cell (up to 18.2 pg P-CTX3C eq./cell, Table 1). A 10-fold lower concentration of 0.1 pg P-CTX3C eq./cell that approximates maximal concentrations produced by the next most toxic species in the Pacific (*G. belizeanus*, Table 1) would require parrotfish feeding on cell densities >150 *Gambierdiscus*/cm^2^ to remain poisonous (Figure 6a). Only two cell densities ≥40 *Gambierdiscus*/cm^2^ have so far been reported from screen assays globally [47], so densities ≥150 cells/cm^2^ would likely only occur during very large blooms. Doubling the depuration half-life to 30 days would require the parrotfish to feed on densities >8 *Gambierdiscus*/cm^2^ producing 1 pg P-CTX3C eq./cell to remain poisonous and >80 *Gambierdiscus*/cm^2^ for 0.1 pg P-CTX3C eq./cell (Figure 6b). However, a slow hypothetical depuration half-life of 1 y could allow the same fish to remain poisonous by feeding on as <1 cell/cm^2^ producing 1 pg P-CTX3C eq. cell, although it would require densities >5 cells/cm^2^ for *Gambierdiscus* producing 0.1 pg P-CTX3C eq./cell (Figure 6d).

Our model suggests that a mildly poisonous 28 cm unicornfish (*N. unicornis*) would have to continuously feed on >1200 *Gambierdiscus*/g macroalgae producing ≤0.1 pg P-CTX3C eq./cell to remain poisonous if it simultaneously depurated CTX with a half-life ≤30 days (Figure 7). Such high *Gambierdiscus* densities are rare, with >85% of reported densities <1000 cells/g [2,91], and these modelled half-life’s are slower than suggested by experimentally derived data for *N. brevistoris* [92] (Table S2, Figure S1). If all unicornfish species rapidly depurate CTX, this could explain patterns of toxicity in Moorea, French Polynesia, where blooms of high CTX-producing *G. polynesiensis* could produce ciguateric unicornfish (Figure 4), although these fish apparently cause less poisonings than many other species [43]. Herbivores are the largest component of Moorea’s local fin fishery [44,85,86], with the major cause of ciguatera being *C. striatus* (28%) [43]. Although the biomass of unicornfishes (including *N. unicornis*) has been reduced by fishing, they continue to be targeted by Moorea’s fishers and taken at high levels relative to their available biomass because of their palatability and commercial value [44,85,86,87,88]. Despite being targeted, unicornfishes were not listed amongst the ciguateric fish species from Moorea by Morin et al. [43], although they are known to cause poisoning throughout French Polynesia [24,38,42,92]. In contrast, other surgeonfishes, *Acanthurus* spp. but especially *C. striatus*, are some of the most abundant taxa available to Moorea’s fishers; however, they are actively avoided, with only small numbers taken for food, presumably because of their high risk of ciguatera [43,44,85,87].

Our model for CTX depuration treats all CTX analogs equally (a single compartment model). However, this may not be the case with different structural forms possibly depurating from fish at different rates. This needs to be experimentally verified, but a slower depuration rate for the P-CTX-1 analogs in parrotfish compared to the P-CTX3C family of toxins (an hypothesis first proposed by Ledreux et al. [115]) could help explain the CTX profiles across French Polynesian food chains shown in Figure 6 in Chinain et al. [24] (an alternate scenario would be for different absorption rates occurring in the fish gut for different structural families of CTX). Chinain et al. [24] described food chains, showing that although the P-CTX3C analogs dominate the CTX profile of *G. polynesiensis* and the three invertebrate species presumably feeding on them, a fourth group of herbivores (parrotfish) were dominated by P-CTX-4 analogs (the precursors of P-CTX-1), with lesser amounts of P-CTX3C analogs. A slower rate of depuration for P-CTX-4 analogs would allow more of these toxins to accumulate into herbivores, as well as the predatory fishes that feed on them, changing the toxin profile relative to the presumed origin (*G. polynesiensis*). This is also consistent with the toxin profile of deep water carnivores in French Polynesia [24,132], and could help explain the development of very high levels of toxicity in some predatory fishes such as moray eels [8,9,24,71,126,133,134]. At present, we have only indirect evidence for slow depuration of CTX from predatory fishes contaminated with the P-CTX-1 family of toxins from moray eels [126] and Spanish mackerel [7]. In contrast, juvenile grouper depurated P-CTX-1, -2 and -3 relatively quickly (≤1 month) [129]; however, juvenile fish may assimilate less CTX into their flesh than larger fish [92,127] or possibly depurate CTX faster [7].

It has been known for some time that ciguatera risk cannot be estimated from only knowing the density of *Gambierdiscus* on a reef [2,24,37,100,101,135,136]. Likely the best metric for estimating ciguatera risk from the base of the food chain would be CTX concentration/cm^2^ of turf algae, or CTX concentration/g macroalgae. The appropriate metric would depend upon the dominant CTX analogs that accumulate in local food chains, and the feeding mode of the herbivore species that poison people or that are preyed upon by higher trophic level fishes to then poison people. Toxicity is ultimately a function of the cumulative load of the CTX analogs occurring within the edible tissues of the fish; however, it would be interesting if this could be related to a CTX feeding unit metric, with possibly 1 unit of P-CTX-1 eq./cm^2^ ≈ 5 units of P-CTX3C eq./cm^2^, based upon the relative potencies of these analogs [2]. Possibly the next best estimate of ciguatera risk would be determining the density of *G. polynesiensis* on reefs in the Pacific, as suggested by Chinain et al. [24]. However, given the broad range of CTX concentrations reported from *G. polynesiensis* (below detection-18.2 pg P-CTX3C eq./cell, [23,25,27,30,63]) and our suggestion that less toxic species of *Gambierdiscus* and *Fukuyoa* could be a ciguatera risk if ingested by herbivorous fish species with high grazing rates relative to their size, some knowledge of the toxicity of the benthic dinoflagellates will likely be necessary, especially with the wide range of CTX concentrations produced by *G. polynesiensis* (Figure 1, [30,63]). This supports our suggestion that ciguatera risk assessments for the Pacific will likely have to be developed at the regional level [22]. The evidence from culture studies is that the ratio of P-CTX3C:P-CTX-4 can vary considerably in *G. polynesiensis* [25,27]. It will be interesting to see if the dominance of P-CTX-1 analogs in ≥third trophic level ciguateric fish from the east coast of Australia [68,69,70] can be explained by a combination of slow depuration of P-CTX-4 relative to P-CTX3C in second or higher trophic level fishes, and biased production of P-CTX-4 relative to P-CTX3C-analogs by *G. polynesiensis* strains in the Coral Sea.

The development of regional risk assessment models for the trophic transfer of CTX through food chains will depend upon the dominant CTX analogs being produced and transferred through local food chains, the trophic level causing human poisoning, regional differences in the way food chains function, and the degree to which human pressures have modified habitats and fish populations. In the cases of the Pacific and Caribbean, not only are the dominant CTX analogs different, but their benthic ecosystems are reported to function in fundamentally different ways [22,137,138,139]. This has arisen in part because of their different biogeographical histories, which has led to a more than 3-fold difference in the diversity of fishes between the Indo-West Pacific and Caribbean regions [137,138,140] and in how different fish assemblages use reef and adjacent habitats [141].

## 3. Conclusions

**Figure 8:**
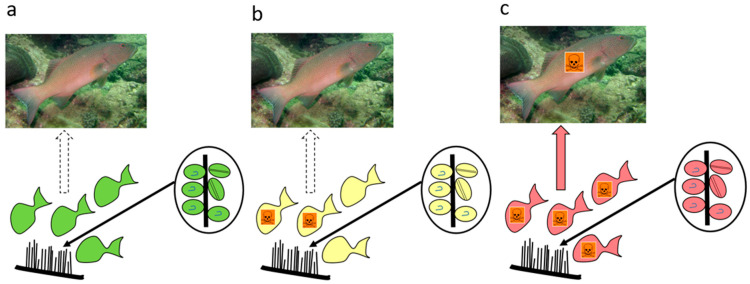
Conceptual three-trophic level food chain for production of ciguateric fishes based upon the concentration of P-CTX3C/*Gambierdiscus* epiphytic on turf algae or macroalgae. Populations of *Gambierdiscus* on turf or macroalgae are grazed by herbivores that are preyed upon by carnivores such as groupers. (**a**) *Gambierdiscus* producing <0.02 pg P-CTX3C eq./cell. Insufficient CTX load can accumulate in this food chain to produce ciguateric 2nd or 3rd trophic level fishes. (**b**) *Gambierdiscus* producing ≥0.02–<~0.1 pg P-CTX3C eq./cell. Insufficient CTX load can accumulate in this food to produce ciguateric 3rd trophic level fishes. Many herbivorous fish species also accumulate insufficient CTX to become poisonous. However, herbivores that graze large areas of turf algae or large amounts of macroalgae relative to their size may accumulate sufficient CTX to become mildly poisonous. (**c**) *Gambierdiscus* producing >~0.1 pg P-CTX3C eq./cell. Sufficient CTX can accumulate in this food chain to produce mildly to highly poisonous 2nd and higher trophic level fishes. (Common coral trout *Plectropomus leopardus* (grouper) image: oris-staff, iNaturalist Australia, Atlas of Living Australia, Catalogue number 109024693, Ala.org.au accessed on 19 September 2025, CC-BY-NC 4.0 (Int).).

Whether herbivorous fish become poisonous depends upon the CTX load they consume and the time they take to accumulate it. The CTX load depends upon the CTX concentration/*Gambierdiscus*, the density of *Gambierdiscus* on turf algae or macroalgae, and the area or weight of algae grazed by fish. We use our food chain model to explore the combinations of *Gambierdiscus* densities, herbivorous fish grazing times, and *Gambierdiscus* CTX concentrations that could produce ciguateric fishes. We suggest the following:

1. Based upon known concentrations of CTX produced by *Gambierdiscus* species (Table 1), most do not produce sufficient CTX to cause ciguatera (Figure 8a). Based upon our model for the Pacific, we suggest that species that produce CTX concentrations ≤0.02 pg P-CTX3C eq./cell have a minimal role in ciguatera.
2. Some *Gambierdiscus* species produce sufficient CTX to potentially accumulate in herbivorous fishes to produce mildly poisonous flesh (Figure 8b). However, it is possible that this scenario is limited to herbivore species that graze large areas/amounts of algae relative to their size. Such mildly poisonous herbivores are unlikely to carry sufficient CTX load that, if preyed upon, would produce poisonous ≥third trophic level fishes (Table S1). Based upon our model for the Pacific, we suggest production of mildly poisonous herbivorous fishes is mostly limited to *Gambierdiscus* species that produce CTX concentrations >0.03 P-CTX3C eq./cell. Apart from *G. polynesiensis*, only *G. belizeanus* and possibly *G. silvae* and *G. australes* are thought to produce >0.03 pg P-CTX3C eq./cell in the Pacific (*G. excentricus* and *G. caribaeus* in the Atlantic).
3. Only high CTX-producing *Gambierdiscus* (>0.1 pg P-CTX3C eq./cell) likely produce sufficient CTX to accumulate in food chains to produce highly toxic ciguateric second trophic level fishes, and weakly to highly toxic ≥third trophic level fishes (Figure 8c). To date, only *G. polynesiensis* in the Pacific (and *G. excentricus* in the Atlantic) is known to be capable of producing >0.1 pg P-CTX3C eq./cell.

The density of epiphytic dinoflagellates on turf and macroalgae varies in both space and time and may often be composed of a mix of species [24,45,46]. In addition, there are many possible permutations that affect the outcome of food chains leading to the production of ciguateric fishes, including fish species, size, grazing rates, etc., introducing many complexities and possibilities not captured by our conceptual model (Figure 8). However, we suggest that it may be possible to infer the broad-scale potential for ciguatera on a reef from simply knowing the *Gambierdiscus* species present on a reef, and the trophic level of the fishes utilized by humans for food from that reef.

Incorporating CTX depuration into our model for the first time using a wide range of potential half-life’s for depuration has allowed the exploration of scenarios where mildly poisonous herbivorous fish ingest CTX at rates that are balanced by depuration. This provides estimates of the *Gambierdiscus*/*Fukuyoa* densities and CTX concentrations required for fish to remain poisonous over time. Unless fish continue to ingest CTX at a rate equal to or greater than the depuration half-life, a poisonous fish will eventually become non-poisonous.

We recognize that our model is based upon linear approximations that may not be fully representative of nature and lack the rigour of error estimates. However, to better understand the development of ciguateric fishes, all the processes occurring within the entire food chain must be considered, from CTX production at scales relevant to the CTX loads bioaccumulated by grazers, trophic transfer efficiencies, and the bioaccumulation of CTX analogs into fish flesh at concentrations capable of poisoning people. Only quantitative food chain models achieve this by integrating the disparate processes from discipline specific studies on dinoflagellates and fish. The development of ciguateric fishes occurs from the interaction of processes across food chains at different temporal and spatial scales. We hope our work provides the framework for the design of better experiments to define the relationships and coefficients necessary to improve ciguatera food chain models. This should improve conceptual understanding of the processes and may help the development of practical risk assessment tools.

## 4. Material and Methods

### 4.1. Models for Bioaccumulation of P-CTX3C into Herbivorous Fish

We adapt our previous models for the bioaccumulation of Pacific-ciguatoxin-1 (P-CTX-1) analogs [33,34] to the bioaccumulation of P-CTX3C toxin equivalents (eq.) into the unicornfish *Naso unicornis* and 4 species of parrotfish, *Scarus frenatus*, *S. niger*, *S. psittacus*, and *Chlorurus sordidus*, in two trophic level food chains. The models are based upon back-calculating the minimum amount of CTX that a 28 cm *N. unicornis*, or a 25 cm parrotfish (total length, TL), must ingest to produce a target flesh concentration that would cause mild poisoning in people (0.5 μg P-CTX3C eq./kg flesh, [2]). The model parameters used to produce the CTX load in fish were calculated (Table 2 and Table 3) based upon the biometric data of the modelled species (Table 4). The model workings were described for parrotfish along with their efficacy and limitations by Holmes and Lewis [34]. The area of turf algae grazed by parrotfish/day was estimated using the equations of Lange et al. [61] (Table 2). We previously used these equations to estimate the areas of turf algae grazed by *S. niger* and *Ch. strongylocephalus* [34], without being aware that Lokrantz et al. [142] had also published equations for the areas grazed by small (<40 cm TL) *S. niger*, *Ch. sordidus*, and *Ch. strongylocephalus* from fish feeding over 3 sites near Zanzibar (although they acknowledge that their equations likely overestimate the areas grazed). We used the Lokrantz et al. [142] equations from the site (Chumbe) that produced the largest area grazed/day for 25 cm *S. niger* and *Ch. sordidus* as confirmation of our model results for the bioaccumulation of P-CTX3C into parrotfish using the equations from Lange et al. [61]. As the estimated area grazed/day by 25 cm *S. niger* and *Ch. sordidus* [142] were less than the estimates calculated by Lange et al. [61], this suggests a model based upon the Lokrantz et al. [142] would require longer feeding times and/or greater *Gambierdiscus* CTX concentrations to produce poisonous 25 cm parrotfish than suggested by our model (Figures S2 and S3), supporting our model results.

The area of turf algae grazed by 18 cm parrotfish was also compared with that of an 18 cm surgeonfish *Ctenochaetus striatus*, with the area grazed by the latter previously estimated by Holmes and Lewis [33].

We estimated the weight of macroalgae grazed/day by a 28 cm *N. unicornis* (282.4 g, Table 4) as a proportion of the body weight of the fish (10–30% body weight, Table 3). There is no data from the field on the weight of macroalgae eaten/day by *Naso* spp., but Clausing et al. [92,127] fed juvenile *N. brevirostris* in aquarium experiments at a rate of 6–10% fish body weight/day. We therefore used consumption of 10% body weight of macroalgae as the minimum potential weight of macroalgae eaten by a 28 cm *N. unicornis*/day (i.e., 28 g macroalgae/day). Herbivorous fish apparently consume an average of ~20% body weight/day, but with some species capable of exceeding 30% and larger fish generally consuming a proportionally smaller percentage [143]. We therefore used consumption of 30% body weight/day as the maximum potential weight of macroalgae consumed by a 28 cm *N unicornis* (i.e., 85 g macroalgae/day).

### 4.2. Modelling Depuration Rates That Balance Ingestion of CTX to Keep Fish Flesh Poisonous

#### 4.2.1. Parrotfish Depuration of CTX

In juvenile fish, rapid somatic growth can dilute the toxin load in the flesh, contributing to an effective reduction in toxin concentration that mimics toxin depuration [7,15,92,127]. This becomes less pronounced as growth slows in older fish. In mature fish, depuration will reduce the toxin load unless CTX is ingested at a rate equal to or greater than the losses from depuration. We consider depuration as any combination of processes that excrete or reduce toxicity in the flesh of fish. We model scenarios to estimate the CTX load a 25 or 40 cm *S. frenatus* would have to ingest to maintain poisonous flesh (0.5 μg P-CTX3C eq./kg flesh or 0.1 P-CTX-1 eq./kg flesh) against CTX loss from hypothetical depuration half-lives (15, 30, 90, and 365 days). We model *S. frenatus* because it grazes the largest area of the 4 parrotfish species (Figure 5) and therefore would ingest the most CTX load from feeding on the same density of *Gambierdiscus* producing the same CTX concentrations. The scenarios we model are combinations of *Gambierdiscus* densities and CTX concentrations that produce a CTX load to balance that lost by each hypothetical depuration rate to maintain mildly poisonous flesh. The model is linear so these estimates for each combination of cell density and CTX concentration are multiples for each calculation.

For depuration of P-CTX3C, the initial model conditions assume the fish has already bioaccumulated CTX to have mildly poisonous flesh (0.5 μg P-CTX3C eq./kg flesh). We then calculate the CTX load from combinations of cell densities (0.1–1000 *Gambierdiscus*/cm^2^) and CTX concentrations (0.1, 1, 4.5, 8.3, or 18.2 pg P-CTX3C eq./cell) that would need to be ingested to double this CTX load in 15, 30, 90, or 365 days, respectively (as if there was no depuration). These combinations of cell densities and CTX concentrations are therefore the same as those necessary to maintain a flesh concentration of 0.5 μg P-CTX3C eq./kg if the fish depurated CTX with half-life’s of 15, 30, 90, or 365 days. We assume that combinations of cell densities and CTX concentrations producing a CTX load greater than that required to balance losses from depuration would result in the P-CTX3C concentrations increasing in the flesh, and similarly, combinations less than this would result in P-CTX3C concentrations decreasing in flesh. Our model produces two outcomes for each possibility depending upon whether 10% or 40% of the toxin load ingested accumulates into flesh (Table 2, Figure 6). The resulting graphs (Figure 6) can therefore be interpreted as consisting of 3 broad areas: (1) An area above the graph lines where the combination of cell densities and toxicities produce flesh with a CTX concentration ≥0.5 μg P-CTX3C eq./kg, labelled as “flesh toxicity increasing”. (2) An area below the graph lines where the toxin concentration in the flesh reduces over time to <0.5 μg P-CTX3C eq./kg, labelled as “flesh toxicity decreasing”. (3) An area in between the graph lines representing the uncertainty in the model for the accumulation of CTX into flesh (10–40%). A limitation of the model is that it effectively treats the transfer of CTX from ingestion to the flesh as happening instantaneously and does not account for the time it would take for P-CTX3C to accumulate into flesh.

The calculations and assumptions above are the same for depuration of P-CTX-1 (Figure 6e–h), except that the mildly poisonous flesh concentration for parrotfish (0.1 μg P-CTX-1 eq./kg) and the *Gambierdiscus* concentrations modelled are lower (0.03, 0.3, 0.6, or 1.6 pg P-CTX-1 eq./cell), consistent with experimental data [27] and the model of Holmes and Lewis [34]. The highest confirmed concentration extracted from *Gambierdiscus* is 0.6 pg P-CTX-1 eq./cell as the sum of the precursor analogs P-CTX-4A and P-CTX-4B [27]. The highest concentration modelled is 1.6 pg P-CTX-1 eq./cell based upon mouse bioassay results, although such a high concentration is yet to be confirmed [33,34].

#### 4.2.2. *Naso unicornis* Depuration of CTX

The depuration scenarios we model for *N. unicornis* estimate the P-CTX3C load a 28 cm unicornfish would have to ingest to maintain poisonous flesh (0.5 μg P-CTX3C eq./kg flesh) against CTX loss from hypothetical depuration half-lives (15, 30 days). The scenarios are calculated similarly to parrotfish, except that we assume 44% of CTX is bioaccumulated into muscle as determined for *N. brevirostris* [92] instead of using a 10–40% range (Table 2), and the *Gambierdiscus* densities ingested are cells/g macroalgae instead of cells/cm^2^ turf algae.

Calculations for the model variables (Table 2, Table 3 and Table 4) were performed using a commercial spreadsheet (Excel, Microsoft). Graphs were constructed using GraphPad Prism 10.6.0. A major limitation of our model is that the uncertainties of the parameters and variables are unknown. It is possible that many variables and their error distributions will differ between species and geographic locations and therefore we use the model to estimate limitations within food chains for bioaccumulation of CTX to produce ciguateric fishes rather than as a model to simulate the most likely pathway for CTX bioaccumulation in each fish species.

## Figures and Tables

**Table 1:** Maximum reported CTX concentrations from *Gambierdiscus* and *Fukuyoa* species from the Pacific Ocean (out of 20 known *Gambierdiscus* and 4 *Fukuyoa* species globally).

Number	Species	Maximum Reported CTX Concentration (pg P-CTX3C eq./cell)	Reference
	*G. polynesiensis* (Cook Islands, Polynesia)	155 pg CTX (attributed only as CTX/cell)	[30]
1	*G. polynesiensis* (Cook Islands, Polynesia)	18.2	[29]
	*G. polynesiensis* (French Polynesia)	8.3	[27]
2	*G. belizeanus*	0.1	[23]
3	*G. australes*	0.03 from Pacific isolate, but 0.5 pg P-CTX-1 eq./cell from Atlantic	[23,48]
4	*G. toxicus*	0.03	[23]
5	*G. carpenteri*	0.03	[24]
6	*G. scabrosus*	0.03	[49]
7	*G. balechii*	0.02	[50]
8	*G. caribaeus*	0.02 from Pacific isolate but up to 0.17 from Atlantic	[11,24]
9	*G. pacificus*	0.008	[24]
10	*G. honu*	0.001	[24]
11	*G. lapillus*	-/trace	[28,51]
12	*G. lewisii*	-/trace	[28,51]
13	*G. holmesii*	-/trace	[6,51]
14	*G. cheloniae*	CTX not yet detected	[28,52]
15	*G. vietnamensis*	CTX not yet detected	[53]
16	*G. silvae*	CTX not quantified from Pacific isolates. Concentrations from outside Pacific suggest ~0.02 pg P-CTX3C eq./cell, 0.08 pg P-CTX-1 eq./cell	[54,55,56]
17	*G. jejeunsis*	CTX not yet detected	
18	*G. bagnisii*	CTX not yet detected	
1	*F. yasumotoi*	CTX not yet detected	[57]
2	*F. paulensis*	CTX not detected from Pacific isolates. Concentrations outside Pacific suggest fg/cell	[58,59]
3	*F. ruetzleri*	CTX not detected from Pacific isolates. Concentrations outside Pacific suggest ~0.03 pg P-CTX3C eq./cell	[55,60]
4	*F. koreansis*	CTX not yet detected	

**Table 2:** Model parameters for herbivorous fish (trophic level 2): parrotfish (*Scarus* or *Chlorurus* spp.) grazing turf algae, and the bluespine unicornfish (*Naso unicornis*) grazing macroalgae that support various densities of *Gambierdiscus* or *Fukuyoa* spp.

Variable	Model Values	Calculations, Assumptions, and Comments
Model target for P-CTX3C concentration in flesh of herbivorous fish	0.5 μg P-CTX3C/kg	0.5 µg P-CTX3C/kg fish is based upon the five-fold lower potency of P-CTX3C relative to P-CTX-1 [2] and is assumed to likely cause mild poisoning in 2 out of 10 people [35]. This CTX concentration is 10-fold more than the US FDA recommended limit of 0.01 μg P-CTX-1 equivalents (eq.)/kg but transformed to P-CTX3C eq., i.e., 0.5 μg P-CTX3C eq./kg.
Flesh (fillet) recovery	Parrotfish: 42% *N. unicornis*: 43%	Parrotfish (42%): Median value of a range of meat recoveries for fillets (40–49%) taken from internet fishing sites for 5 species of *Scarus* spp. [34]. *N. unicornis* (43%): We can find no data for filet recovery from fishers, so we have estimated a meat recovery of 43% based upon the average weight of muscle recovered from juvenile *N. brevirostris* (40%, 47%) [92]. This may be an overestimate for meat recovery from filets.
Flesh (fillet) CTX burden	Parrotfish: A range is calculated between 10 and 40% of the CTX load ingested by parrotfish. *N. unicornis*: 44%	Parrotfish: Flesh estimated to accumulate between 10–40% of the toxin load of the fish based upon Caribbean pinfish [128]. Clausing et al. [92] recently reported a slightly higher relative proportion of CTX retained in the muscle of the unicornfish *N. brevirostris* (44%). *N. unicornis*: Based on the 44% retention of CTX reported for *N. brevirostris* [92].
Fish CTX load (μg)	Calculated depending upon fish weight (Table 4)	Based upon a 43% transfer rate [128]
Daily grazing rates. Parrotfish: m^2^/d on turf algae. *N. unicornis*: g macroalgae/day.	Parrotfish: Calculated from annual grazing rates (m^2^/y) depending upon species and fish total length (TL, cm). *N. unicornis*: Estimated grazing rate as g macroalgae/day based upon the fish consuming a percentage of its body weight.	Parrotfish: Annual grazing rates (m^2^/y) calculated using equations derived by Lange et al. [61]: *S. niger* = 0.0367(TL^2.2^), *S. frenatus* = 0.0138(TL^2.439^), *S. psittacus* = 0.0004(TL^2.986^), *Chlorurus sordidus* = 0.0433(TL^2.209^). Lokrantz et al. [142] also derived equations for the area grazed by *S. niger* and *Ch. sordidus* for fish observed feeding at 3 sites near Zanzibar. The equations for the Chumbe site produced the largest area grazed (cm^2^/min): *S. niger* = 0.00002(TL^3.66^), *Ch. sordidus* = 0.0001(TL^3.09^). Lokrantz et al. [142] suggest that their equations likely overestimate the areas grazed. *N. unicornis*: 10–30% body weight of macroalgae [92,127,143]
The time parrotfish and *N. unicornis* spend grazing on turf algae each day	9 h	Algae is a low-energy food source requiring many of the herbivores that rely on it for nutrition to feed almost continuously during daylight hours [108,144,145,146,147] and 9 h is consistent with the daily feeding times we used previously for parrotfish and surgeonfish on the Great Barrier Reef [33,34]. We have modified the daily feeding from 12 h used by Lange et al. [61] for parrotfish feeding close to the equator in the Maldives and Chagos Archipelago. However, feeding duration likely varies throughout the day, between seasons and with latitude
The efficacy of the fish bite to remove and ingest *Gambierdiscus* from algae.	Parrotfish: 90% *N. unicornis*: 100%	Parrotfish: This rate is an assumption as there are no data available but is unlikely to be 100% as the bite is acting on a surface covered with turf algae. However, as scraping and excavator parrotfish are targeting microorganisms for nutrition [95,148,149,150] we assume the efficiency to be high. *N. unicornis*: The model assumes that the fish bite removes pieces of algae with attached epiphytic dinoflagellates. We have assumed 100% bite efficiency but recognize that this is likely an overestimation.

**Table 3:** Model parameters for trophic level 1: *Gambierdiscus* epiphytic upon turf algae grazed by parrotfish or macroalgae grazed by unicornfish.

Variable	Model Values	Calculations, Assumptions, and Comments
The transfer rate for CTX between trophic level 1 and 2	43%	Based upon an average net CTX assimilation of 43% in pinfish [128], also see Holmes and Lewis [32,33,34]. This term accounts for CTX losses between trophic levels. This transfer efficiency is similar to that reported for CTX from *G. polynesiensis* into mullet (42%, [115]). The actual transfer rates for the modelled species are not known
P-CTX3C concentrations produced by *Gambierdiscus* and consumed by herbivorous fish. These concentrations are varied depending upon the scenario being explored	0.01–155 pg P-CTX3C eq./cell	Scenarios for parrotfish and *N. unicornis* explore a range of potential toxin concentrations based upon concentrations determined experimentally. The maximum concentration is assumed to be 18.2 pg P-CTX3C eq./cell from *G. polynesiensis* isolated from Rarotonga in the Cook Islands, Polynesia [29] although Rhodes et al. [30] suggest this isolate had earlier produced 155 pg CTX/cell. The maximum known concentration from French Polynesian *G. polynesiensis* is 8.3 pg P-CTX3C eq./cell [27]. Depuration scenarios for parrotfish include comparisons with *Gambierdiscus* producing hypothetical P-CTX-1 concentrations between 0.03–1.6 pg P-CTX-1 eq./cell. 1.6 pg P-CTX-1 eq./cell is a hypothetical concentration based upon mouse bioassay of *Gambierdiscus* strains isolated from Platypus Bay, and the Great Barrier Reef, Australia [32,33]. All calculations for these P-CTX-1 scenarios were as per Holmes and Lewis [34].
*Gambierdiscus* densities on turf algae or macroalgae	Turf algae: 0.1–10,000 cells/cm^2^ Macroalgae: 1–10,000 cells/g	Hypothetical range of cell densities of CTX-producing *Gambierdiscus* epiphytic on turf algae or macroalgae. Parrotfish: Cell densities on turf algae (cells/cm^2^) are compared with ranges reported from 24 h benthic screen assays ([47] and references therein). We are not aware of any reports of cell densities ≥1000 cells/cm^2^. ~1 cell/cm^2^ is the median of a global range on screen assays [47]. *N. unicornis*: Cell densities (cells/g macroalgae) are compared with ranges from the literature [2,91].

**Table 4:** Calculations for lengths and weights of modelled fish species.

Scraping Parrotfish Species	Common/Local Name	Maximum Total Length (cm) [107,151]	Weight (g)–Total Length (TL, cm) Relationships	Reference for Weight–Length Relationships
*Scarus frenatus*	Sixband parrotfish	47	Weight (g) = 0.0366∙TL^2.816^	[105]
*S. niger*	Swarthy parrotfish	40	Weight = 0.041∙TL^2.75^	[105]
*S. psittacus*	Palenose parrotfish	43	Weight (g) = 0.0189∙TL^3.03^ FishBase calculator for American Samoa	[107]
**Excavator parrotfish species**				
*Chlorurus sordidus* Note: From Indian Ocean. The fish previously labelled *Ch*. (*Scarus*) *sordidus* from the Pacific is likely *Ch. spilurus* [151]	Bullethead parrotfish	40	Weight (g) = 0.109∙TL^2.48^	[105]
**Surgeonfish species**				
*Ctenochaetus striatus*	Lined surgeonfish	26	Weight (g) = 0.0137∙TL^3.083^ FishBase calculator for Réunion Is.	[107]
*Naso unicornis*	Bluespine unicornfish	74	Weight (g) = 0.0329∙FL^2.85^ FishBase calculator for American Samoa. Fork Length (FL) = 0.857∙TL	[107]
**Predator species**				
*Plectropomus leopardus*	Common coral trout (grouper)	120 (23.6 kg)	Model a 2 kg fish	

## Data Availability

The original contributions presented in this study are included in the article/Supplementary Material. Further inquiries can be directed to the corresponding author.

## Supplementary Materials

The following supporting information can be downloaded at https://www.mdpi.com/article/10.3390/toxins17110526/s1: Table S1: Scenario calculating how many mildly poisonous prey fish (0.5 μg P-CTX3C eq./kg flesh) a 2 kg grouper would have to consume to develop mildly poisonous flesh. Calculations follow Holmes and Lewis [33]. Table S2: Applying a 15-day half-life for depuration of ingested ciguatoxin (CTX) to the weight-adjusted feeding of *Gambierdiscus polynesiensis* (89 *G. polynesiensis*/g body weight/day) to juvenile unicornfish (*Naso brevirostris*) [92] results in 28.9% of residual CTX after 120 days. This approximates the 26% residual CTX experimentally detected after 120 days by Clausing et al. [92] but suggests that the actual depuration half-life is slightly quicker than 15 days. For simplicity, the calculations are based on ingested CTX without incorporating the losses for each trophic transfer in the model (43%, Table 3). Figure S1: Data from Table S2 plotted as cumulative P-CTX3C load eaten by *Naso brevirostris* for scenarios assuming no CTX depuration, and depuration with a half-life of 15 days. P-CTX3C load based upon 4.5 pg P-CTX3C eq./*Gambierdiscus polynesiensis* [92]. Clausing et al. [92] found that only 26% of the CTX load ingested by *N. brevirostris* was retained after 120 days of weight-adjusted feeding of *G. polynesiensis*. Figure S2: Comparison of modelled grazing times (days) for a 25 cm *Scarus niger* calculated from the equations for area grazed by Lange et al. [61] (solid line) and Lokrantz et al. [142] (dashed line) to accumulate a flesh concentration of 0.5 μg P-CTX3C eq. from feeding on turf algae supporting different densities of *Gambierdiscus* producing 0.1–18.2 pg P-CTX3C eq./cell. The model produces two outcomes for each cell concentration depending upon whether 10% or 40% of the toxin-load ingested accumulates into flesh: only 40% (maximum potential toxicity) plotted for each P-CTX3C concentration. Scenarios are based on bioaccumulation of CTX without depuration. Figure S3: Comparison of modelled grazing times (days) for a 25 cm *Chlorurus sordidus* calculated from the equations for area grazed by Lange et al. [61] (solid line) and Lokrantz et al. [142] (dashed line) to accumulate a flesh concentration of 0.5 μg P-CTX3C eq. from feeding on turf algae supporting different densities of *Gambierdiscus* producing 0.1–18.2 pg P-CTX3C eq./cell. The model produces two outcomes for each cell concentration depending upon whether 10% or 40% of the toxin-load ingested accumulates into flesh: only 40% (maximum potential toxicity) plotted for each P-CTX3C concentration. Scenarios are based on bioaccumulation of CTX without depuration.
