# Supplementary material for: A General Food Chain Model for Bioaccumulation of Ciguatoxin into Herbivorous Fish in the Pacific Ocean Suggests Few Gambierdiscus Species Can Produce Poisonous Herbivores, and Even Fewer Can Produce Poisonous Higher Trophic Level Fish
Source: Toxins (Basel). 2025 Oct 25;17(11):526. doi: 10.3390/toxins17110526 (PMC12656673; doi:10.3390/toxins17110526)
Supplement: Supplementary file 1 [file toxins-17-00526-s001.zip › toxins-3916122-supplementary.pdf]

A General Food Chain Model for Bioaccumulation of Ciguatoxin into Herbivorous Fish in the Pacific Ocean Suggests Few *Gambierdiscus* Species Can Produce Poisonous Herbivores, and even Fewer Can Produce Poisonous Higher Trophic Level Fish

Michael J. Holmes and Richard J. Lewis

Table S1. Scenario calculating how many mildly poisonous prey fish (0.5 µg P-CTX3C eq./kg flesh) a 2 kg grouper would have to consume to develop mildly poisonous flesh. Calculations follow Holmes and Lewis [33].

Calculating	Result of calculation	Source/Reference for calculation
**P-CTX load of mildly poisonous 2 kg grouper**		
Flesh (muscle) of 2 kg grouper (*Plectropomus leopardus*) (50% meat recovery)	1 kg	[33]
P-CTX load to produce a concentration of 0.5 µg P-CTX3C eq./kg in 1 kg flesh	0.5 µg P-CTX3C eq.	
P-CTX load for the 2 kg fish based upon flesh assimilating 10% to 40% of toxin load ingested	1.3-5 µg P-CTX3C eq.	[128]
**Estimating number of mildly poisonous 100 g prey fish (e.g. unicornfish *Naso unicornis*) consumed to produce mildly poisonous grouper. Optimal meal size for grouper = 5% body weight [120]**		
Flesh (muscle) of 100 g *N. unicornis*	43 g	Table 2, [92]
P-CTX load to produce a concentration of 0.5 µg P-CTX3C eq./kg in 43 g flesh	21.5 ng P-CTX3C eq.	
P-CTX load for the 100 g fish based upon flesh assimilating 44% of toxin load ingested	48.9 ng P-CTX3C eq.	Table 2, [92]
P-CTX load for the 100 g fish incorporating toxin loss during trophic transfer (predation/consumption/digestion) to	21.0 ng P-CTX3C eq.	[128]

grouper. i.e., P-CTX trophic assimilation efficiency of 43%		
Number of 100 g *N. unicornis* a 2 kg *P. leopardus* would need to consume to produce a flesh concentration of 0.5 μg P-CTX3C eq./kg. i.e., P-CTX3C load for 2 kg grouper (1.3-5 μg)/P-CTX3C load of 100 g unicornfish (21 ng).	60-238	
**Estimating the time for a 2 kg grouper to consume 60-238 mildly poisonous prey based upon grouper feeding 3 times/week (Johansen et al. [120] suggest feeding 2-3 times/week). This equates to the grouper consuming an average of 2.1% bodyweight/day. Scenario calculations in the absence of CTX depuration and fish growth.**		
Weeks to prey on 60 *N. unicornis*	20 weeks (140 days)	
Weeks to prey on 238 *N. unicornis*	80 weeks (560 days)	

Table S2. Applying a 15-day half-life for depuration of ingested ciguatoxin (CTX) to the weight-adjusted feeding of *Gambierdiscus polynesiensis* (89 *G. polynesiensis*/g body weight/day) to juvenile unicornfish (*Naso brevirostris*) [92] results in 28.9% of residual CTX after 120 days. This approximates the 26% residual CTX experimentally detected after 120 days by Clausing et al. [92] but suggests that the actual depuration half-life is slightly quicker than 15 days. For simplicity, the calculations are based on ingested CTX without incorporating the losses for each trophic transfer in our model (43%, Table 3).

**Day**	**15**	**30**	**45**	**60**	**75**	**90**	**105**	**120**	**Total**	**% Remaining after 120 days**
Average weight of the fish (g)	38.9	41.4	44.0	46.8	49.8	52.9	56.3	59.9		
**A.** Total ingested burden on each 15th day (i.e. fish weight · 89 cells · 15	51,957.8	55,253.5	58,758.2	62,485.3	66,448.8	70,663.6	75,145.9	79,912.4	**520,625.5**	**100%**

days). Summed total is the same as no CTX depuration										
**B.** Proportion of CTX remaining on each 15th day for 120 days feeding assuming a depuration half-life of 15 days	0.007813	0.015625	0.03125	0.0625	0.125	0.25	0.5	1		
Proportion of CTX remaining of ingested burden after each 15th day (i.e. **A·B**). Equivalent to CTX depuration half-life of 15 days.	405.9	863.3	1836.2	3905.3	8306.1	17,665.9	37,572.9	79,912.4	**150,468.2**	**28.9%**

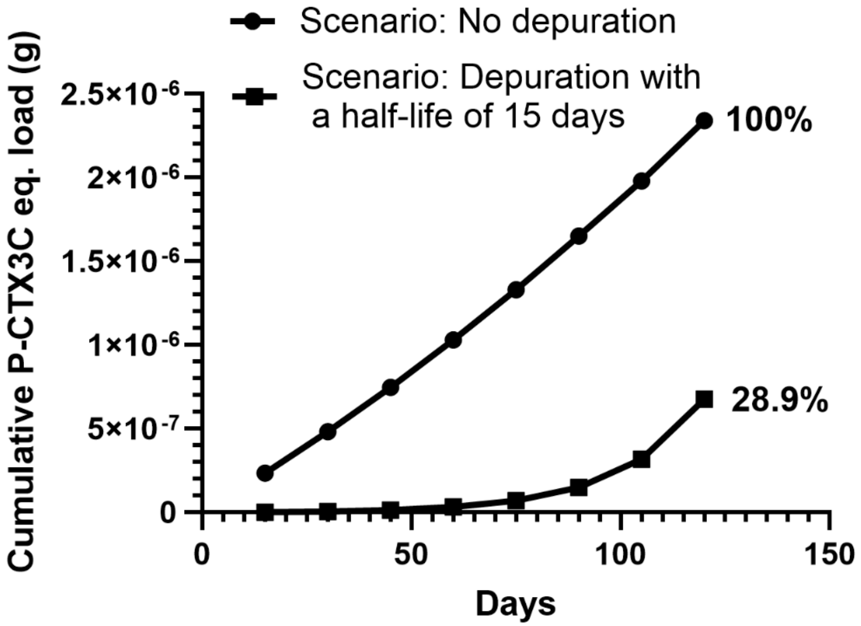

Figure S1. Data from Table S2 plotted as cumulative P-CTX3C load retained by *Naso brevirostris* after 120 days for scenarios assuming no CTX depuration, and depuration with a half-life of 15 days. P-CTX3C load based upon 4.5 pg P-CTX3C eq./*Gambierdiscus polynesiensis* [92]. Clausing et al. [92] found that only 26% of the CTX load ingested by *N. brevirostris* was retained after 120 days of weight-adjusted feeding of *G. polynesiensis*.

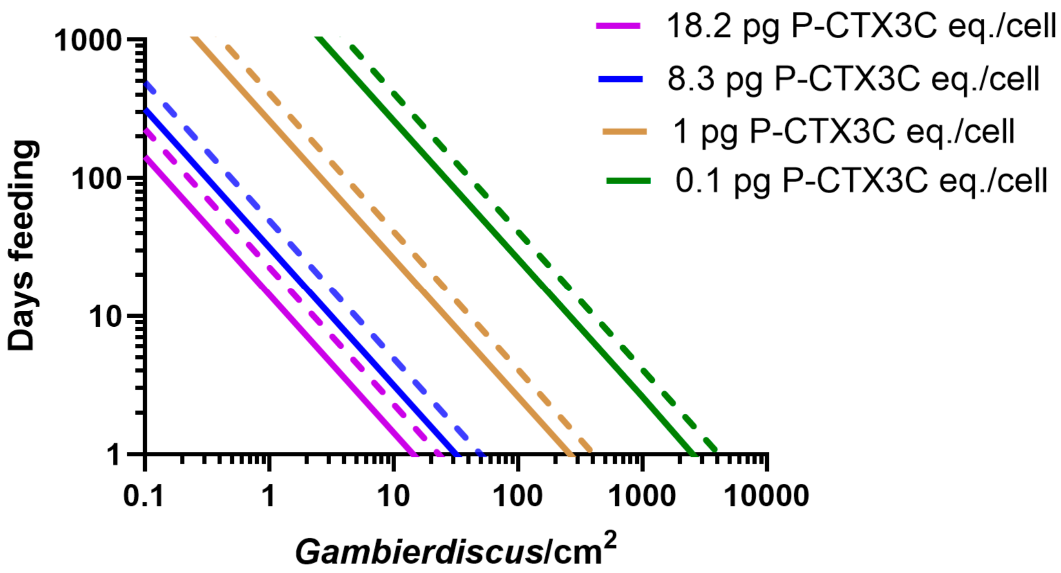

Figure S2. Comparison of modelled grazing times (days) for a 25 cm *Scarus niger* calculated from the equations for area grazed by Lange et al. [61] (solid line) and Lokrantz et al. [142] (dashed line) to accumulate a flesh concentration of 0.5 µg P-CTX3C eq. from feeding on turf algae supporting different densities of *Gambierdiscus* producing 0.1-18.2 pg P-CTX3C eq./cell. The model produces two outcomes for each cell concentration depending upon whether 10% or 40% of the toxin-load ingested accumulates into flesh: only 40% (maximum potential toxicity) plotted for each P-CTX3C concentration. Scenarios are based on bioaccumulation of CTX without depuration.

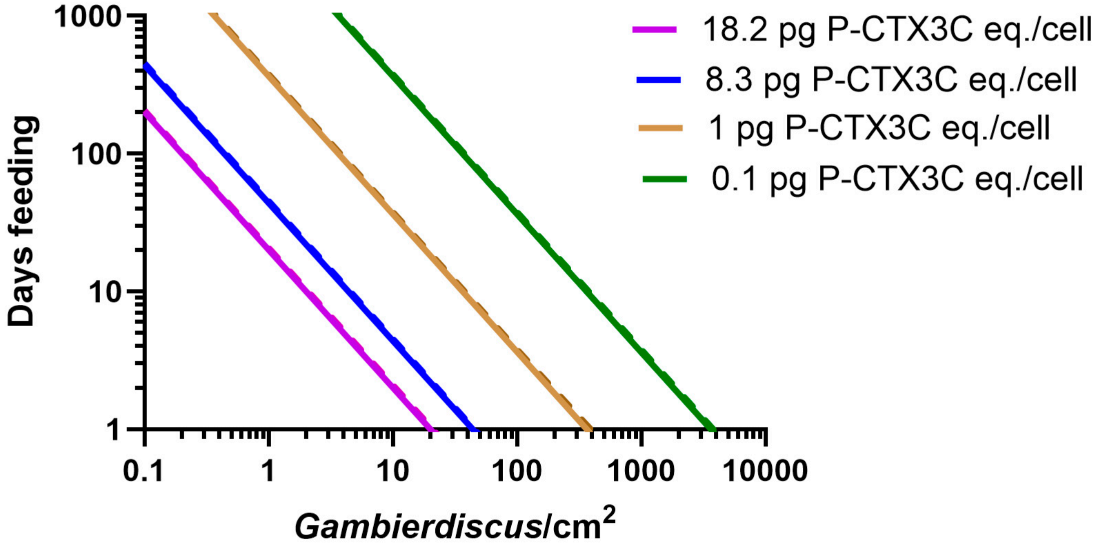

Figure S3. Comparison of modelled grazing times (days) for a 25 cm *Chlorurus sordidus* calculated from the equations for area grazed by Lange et al. [61] (solid line) and Lokrantz et al. [142] (dashed line) to accumulate a flesh concentration of 0.5 µg P-CTX3C eq. from feeding on turf algae supporting different densities of *Gambierdiscus* producing 0.1-18.2 pg P-CTX3C eq./cell. The model produces two outcomes for each cell concentration depending upon whether 10% or 40% of the toxin-load ingested accumulates into flesh: only 40% (maximum potential toxicity) plotted for each P-CTX3C concentration. Scenarios are based on bioaccumulation of CTX without depuration.
